# Supplementary material for: Seminoma and Embryonal Carcinoma Footprints Identified by Analysis of Integrated Genome-Wide Epigenetic and Expression Profiles of Germ Cell Cancer Cell Lines
Source: PLoS One. 2014 Jun 2;9(6):e98330. doi: 10.1371/journal.pone.0098330 (PMC4041891; doi:10.1371/journal.pone.0098330)
Supplement: File S1 — ZIP file containing DMRforPairs output for significant regions. Please start from the html files. (ZIP) [file pone.0098330.s008.zip › figures/22759.pdf]

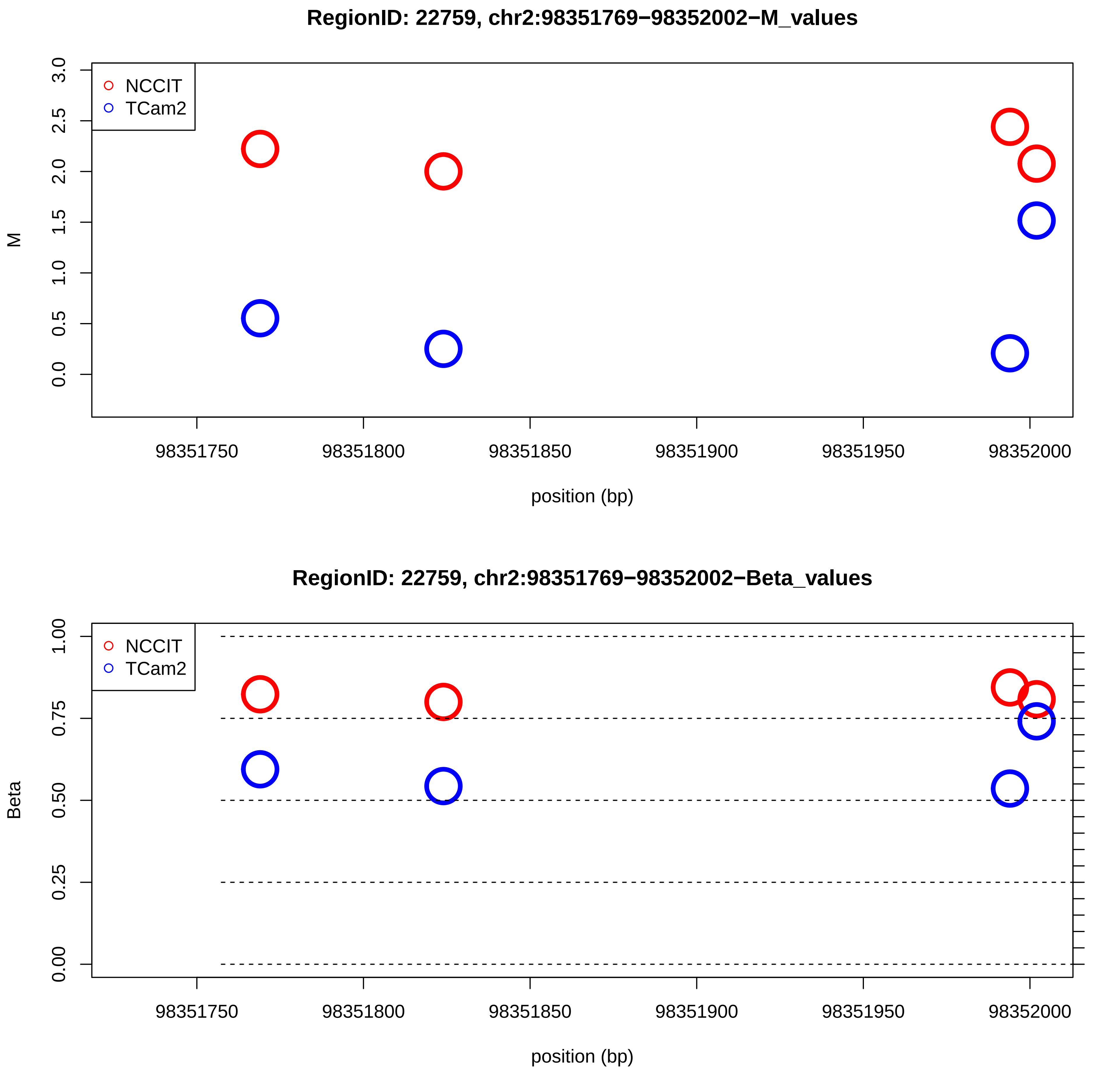
RegionID: 22759, chr2:98351769−98352002−M_values
NCCIT
TCam2
M
0.0
0.5
1.0
1.5
2.0
2.5
3.0
98351750
98351800
98351850
98351900
98351950
98352000
position (bp)
RegionID: 22759, chr2:98351769−98352002−Beta_values
NCCIT
TCam2
Beta
0.00
0.25
0.50
0.75
1.00
98351750
98351800
98351850
98351900
98351950
98352000
position (bp)
